# Sex differences in the impact of lower respiratory tract infections on older adults’ health trajectories: a population-based cohort study

**DOI:** 10.1186/s12879-024-10131-7

**Published:** 2024-11-01

**Authors:** Ahmad Abbadi, Giorgi Beridze, Eleana Tsoumani, Agnes Brandtmüller, Merle K Hendel, Stina Salomonsson, Amaia Calderón-Larrañaga, Davide L. Vetrano

**Affiliations:** 1 Aging Research Center, Department of Neurobiology, Care Sciences and Society, Karolinska Institutet and Stockholm University, Stockholm, Sweden; 2 Center for Observational and Real-World Evidence, MSD, Athens, Greece; 3 Center for Observational and Real-World Evidence, MSD, Budapest, Hungary; 4 Center for Observational and Real-World Evidence, MSD, Stockholm, Sweden; 5 Stockholm Gerontology Research Center, Stockholm, Sweden; 6 Department of Medical Epidemiology and Biostatistics, Karolinska Institutet, Nobels väg 12A, 171 65 Solna, Sweden

**Keywords:** Lower respiratory tract infections, Pneumonia, Older adults, Geriatric Health Assessment, Sex stratification

## Abstract

**Background:**

Lower respiratory tract infections (LRTIs) are a major global health concern, particularly among older adults, who have an increased risk of poorer health outcomes that persist beyond the acute infectious episode. We aimed to investigate the mid-term (up to 7 years) and long-term (up to 12 years) effects of LRTIs on the objective health status trajectories of older adults, while also considering potential sex differences.

**Methods:**

Cohort data of adults aged ≥ 60 years from the Swedish National study of Aging and Care in Kungsholmen (SNAC-K) collected between 2001 and 2016 was analyzed. Information on LRTIs was obtained from the Swedish National Patient Register, and objective health status was assessed using the Health Assessment Tool (HAT) which incorporates indicators of mild and severe disability, cognitive and physical functioning, and multimorbidity. The LRTI-exposed and -unexposed participants were matched using propensity score matching based on an expansive list of potential confounders. Mixed linear models were used to analyze the association between LRTIs and changes in HAT scores.

**Results:**

The study included 2796 participants, 567 of whom were diagnosed with a LRTI. LRTIs were independently associated with an excess annual decline of 0.060 (95% CI: -0.107, -0.013) in the HAT score over a 7-year period. The associations were stronger among males, who experienced an excess annual decline of 0.108 (95% CI: -0.177, -0.039) in up to 7-years follow-up, and 0.097 (95% CI: -0.173, -0.021) in up to 12-years follow-up. The associations were not statistically significant among females in either follow-up period.

**Conclusion:**

LRTIs, even years after the acute infectious period, seem to have a prolonged negative effect on the health of older adults, particularly among males. Preventative public health measures aimed at decreasing LRTI cases among older adults could help in preserving good health and functioning in old age.

**Supplementary Information:**

The online version contains supplementary material available at 10.1186/s12879-024-10131-7.

## Background

Lower respiratory tract infections (LRTIs) pose a significant global health challenge, ranking as the leading cause of mortality among communicable diseases and the sixth leading cause of death across all age groups [1, 2]. In Sweden, LRTIs were the eighth leading cause of death in 2019 [3]. Notably, the incidence of LRTIs has been on the rise among adults aged 70 years and above worldwide [1, 2], as well as among individuals aged 65 and older in Sweden [4]. Moreover, sex differences have been observed in relation to LRTIs, with males, particularly those aged 70 years and above, having a higher incidence [4, 5] and increased immediate mortality following infection [5].

The increased susceptibility of older adults to LRTIs can be attributed to various factors associated with the aging process, including physiological changes (e.g., reduced lung capacity), immunological alterations, and comorbidity burden (e.g., congestive heart failure or diabetes) [6, 7]. Aging-related factors not only impact LRTI risk and prognosis, but also hinder the individual’s ability to recover after an LRTI episode, which often entails a prolonged recovery period before full functionality is regained [8–11]. In some cases, individuals, particularly those with frailty, may experience longer recovery times or never fully regain their previous levels of functioning [12].

Immediate effects of LRTIs on health and health outcomes are readily described [2, 5, 13]. However, long-term consequences of LRTI vary according to age of onset and severity, as early-life LRTI infections are associated with altered immune response, and increased risk of asthma [14]. In contrast in older adults, recent evidence suggests that the effect of LRTIs can persist beyond the infectious episode, as reversible cognitive decline was described in up to 2.5-years following the infectious episode [11]. Moreover, following a single LRTI episode warranting hospitalization or specialist care, the risk of mortality can remain elevated for up to 16-years, and healthcare utilization for up to 5-years from the LRTI episode [15]. These observed effects were higher among males [15].

The United Nation projects that globally from 2021 to 2050 a doubling of the population aged 65 years or older (1.1 billion to 2.1 billion persons) will occur mainly due to aging in low- and middle-income country, in addition to the ongoing aging in high-income countries [16]. Accurate monitoring and timely prevention of health status decline in older individuals following acute stressors such as LRTIs are crucial for providing personalized and impactful care, while also mitigating the burden on healthcare systems [17]. While research has traditionally primarily focused on the presence and management of chronic conditions when evaluating older adults’ health, a growing body of evidence emphasizes the importance of incorporating multiple dimensions (e.g., cognitive, physical, and social) of aging to obtain a more comprehensive and accurate understanding of health in old age [18, 19]. It has been shown that multiple geriatric indicators considered together have stronger discriminative capacities than individual indicators alone [20]. Thus, objective multidimensional comprehensive tools, such as the Health Assessment Tool (HAT), a reliable and internally and externally validated pragmatic holistic index, can aid in clinical appraisal and decision-making by capturing complex changes across various health domains in old age [21–23].

Although it is well recognized that objective health status declines with age [24], certain acute events, such as LRTI, may potentially accelerate this decline by exerting an independent effect. Moreover, even though the immediate negative effects of LRTIs on health status are well described, it is less clear whether such effects persist or are present at longer follow-up periods that extend beyond the infectious period or the period of increased healthcare utilization (up to 5 years) that follows the infectious episode [15]. Therefore, in this study we aimed to: (1) study the mid-term (up to seven years) and long-term (up to 12 years) effects of a single-episode of LRTI on older adults’ health trajectories, and (2) examine potential sex differences in the impact of single-episode of LRTIs on such trajectories.

## Methods

### Study design, population and data source

This population-based cohort study is based on data from the Swedish National Study of Aging and Care in Kungsholmen (SNAC-K). SNAC-K consists of randomly sampled individuals aged 60 years and above living in Kungsholmen, Stockholm. A total of 3363 individuals (73.3% response rate) attended the baseline study visit that took place between 2001 and 2004, with follow-up waves every six years for the younger group (< 78 years) and every three years for the older group (≥ 78 years). Participants completed questionnaires and underwent comprehensive examinations by a physician, nurse, and psychologist at each study visit. Further information regarding the design of SNAC-K is available elsewhere [25]. Participants were linked to the Swedish National Patient Register (NPR), where information on inpatient and specialist outpatient diagnoses is registered using the International Classification of Diseases, 10th revision (ICD-10) codes [26, 27]. The NPR has high completion and validity [26, 28]. The present study includes 2796 participants with complete information on all covariates of interest. They were followed up until wave 5 (2013–2016), corresponding to the latest available NPR data at the time of data analysis.

### Exposure

The exposure of interest in this study was LRTI, which was identified in the NPR using ICD-10 codes J09-J18 and J20-2. Table S1in Additional file 1 lists the ICD-10 codes used to define the LRTI episodes and their corresponding descriptions. Participants who received at least one LRTI diagnosis during the follow-up period were considered LRTI-exposed at the time of first diagnosis regardless of the number of future LRTI episodes, whereas those who were not diagnosed with LRTI during the entirety of the follow-up were considered LRTI-unexposed. The date of first LRTI episode was used as exposure time for LRTI-exposed participants. Since the NPR only includes data from inpatient records and specialized outpatient care [26, 27], the LRTI cases are considered to reflect moderate and severe LRTI cases that warranted either hospitalization or specialized outpatient healthcare and do not include cases that did not require care or were exclusively managed in primary care.

### Outcome

Change in objective health status over time was considered the outcome of interest. Objective health status was operationalized using the HAT, which is a multidimensional, externally validated, holistic geriatric health assessment tool built using nominal response models [22, 23, 29]. It encompasses information from five geriatric indicators capturing five health dimensions: personal activities of daily living (P-ADL) for severe disability, instrumental activities of daily living (I-ADL) for mild disability, Mini-Mental State Examination (MMSE) for cognitive function, gait speed for physical function, and count of chronic diseases for disease burden [22]. P-ADL and I-ADL were assessed using a questionnaire administered by a trained nurse. Gait speed was assessed using the 6-meter test, and the 2.44-meter test for those with walking difficulties. Count of chronic diseases was based on the operationalization by Calderón-Larrañaga et al. [30], and disease diagnoses were recorded by SNAC-K physicians based on self-reports during cohort follow-ups, medical charts, medication review, register data, and laboratory findings. HAT scores range from 0 (poor health) to 10 (good health), with the following clinically relevant thresholds: 0-1.9 = severe functional dependence; 2-4.9 = mild functional dependence; 5-6.9 = compromised physical functioning with multimorbidity and medium cognitive functioning; 7-8.9 = light decreased physical or cognitive functioning with some morbidities; 9–10 = good physical functioning and morbidity status [31]. The HAT was developed using the aforementioned five health indicators, with optimal cut-offs determined by nominal response models (NRm) against a latent health variable. Weights for each indicator category were derived by regressing the NRm test characteristic curves on the health indicators. The final HAT scores were then transformed into a continuous 0–10 scale. Further information, including the detailed code of transforming the values of each health indicator into the final score is described elsewhere [23]. In previous studies, HAT outperformed other frailty indices and health indicators in predicting short- and long-term mortality [32], hospitalization [32], and social and medical care use [29]. Information on the different HAT components was collected at each SNAC-K study visit. The statistical method of constructing HAT has been validated [21] and replicated [23], and external validation of the HAT has been done in three other aging cohorts in Sweden [23]. Since the HAT is assessed at the fixed intervals of the SNAC-K follow-ups, participants had one-to-two follow-up assessments in the mid-term follow-up (up to 7 years), and two-to-five follow-up assessments in the long-term follow-up (up to 12 years).

### Covariates

The covariates considered in the analyses included three types of potential confounders based on literature-described variables [33–35] : (1) sociodemographic characteristics: age (continuous), sex (male/female), highest level of education attained based on formal education years (elementary (up to 9th grade), high school (10th to 12th grade), and university or higher), living arrangement (independent/ group living), and civil status (married, widowed, unmarried, divorced); (2) Lifestyle factors: smoking status (never, former, current), alcohol intake (never/occasional, light/moderate, heavy), and body mass index (BMI, continuous); (3) Clinical characteristics: count of medications taken (continuous, each active component counts as one medication), and presence of the following chronic conditions; asthma, atrial fibrillation, cerebrovascular disease, chronic kidney disease, chronic liver disease, chronic obstructive pulmonary disease (COPD), cancer, diabetes, heart failure, hypertension, and ischemic heart disease.

### Statistical analysis

Demographic information was summarized using means and standard deviations (SD) for continuous variables and counts with percentages for categorical variables. Chi-square, ANOVA, t-test, and Kruskal-Wallis tests were used to assess differences in demographic variables between the LRTI-exposed and -unexposed groups, as appropriate.

### Construction of the dataset and index date

For exposed participants, the index date was set as the date of their first recorded LRTI-related diagnosis. Due to the potential bias from having different inclusion time points from having the LRTI-exposed participants included at a later point in time, a simulation of LRTI exposure date was performed for LRTI-unexposed participants. This was achieved by assigning them a random index date within the entire follow-up period. This approach aimed to reduce potential bias and minimize the age discrepancies between the exposed and unexposed groups. Henceforth, the most recent HAT measure was carried forward to the index date for each participant.

### Matching using propensity score

To further minimize potential biases arising from the observational study design, each exposed participant was matched to three unexposed participants (i.e., one LRTI exposed: three no-LRTI exposed) based on their propensity score. In doing so, we aimed to make the exposed and unexposed groups more balanced in terms of important confounders. Each unexposed participant could only be matched to one exposed participant, and optimal matching was used. The following covariates were used in the propensity score generation: sex, age, living arrangement, civil status, education, smoking status, alcohol intake, BMI, count of medication taken, count of chronic diseases, gait speed, MMSE, I-ADL, P-ADL, asthma, atrial fibrillation, cerebrovascular disease, chronic kidney disease, chronic liver disease, COPD, cancer, diabetes, heart failure, hypertension, and ischemic heart disease. The covariates used in the matching were based on the follow-up visit immediately preceding the index date. This method has been used previously and is described in detail elsewhere [11, 15].

### Mixed linear models

Mixed linear models with random intercepts and slopes were used to analyze the association between first-episode of LRTI exposure and changes in the HAT scores over time. An interaction term between LRTI exposure and follow-up time was included to quantify the annual differences in the HAT score slopes between individuals with and without an LRTI diagnosis. Two models were computed: one considering the long-term effects using data from all available post-LRTI assessments (up to 12 years of follow-up), and one considering mid-term effects based on the first post-LRTI assessment (up to 7 years of follow-up). Considering that the propensity score matching process was insufficient in accounting for confounding, both models were additionally adjusted for the covariates used in the matching. Stratified analyses were conducted to examine potential sex differences in the observed associations. All analyses were performed using STATA 16.1 (StataCorp, College Station, TX, USA).

### Sensitivity analysis

The analyses were repeated after restricting the exposure group to those with pneumonia diagnoses only. Pneumonia cases were identified using J9-J18 and/or J20-21 ICD-10 codes as illustrated in in Table S1 in Additional file 1.

Strengthening the reporting of observational studies in epidemiology (STROBE) checklist is available in Additional file 2.

## Results

### Baseline characteristics of the study population

Of the 2,796 individuals included in the study, 567 (20.3%) were diagnosed with an LRTI during the follow-up period. They were matched to 1,701 participants who did not receive an LRTI diagnosis. Table 1 provides an overview of the baseline sociodemographic, clinical, and lifestyle characteristics of participants stratified by LRTI diagnosis. On average, individuals diagnosed with LRTIs were older, had fewer years of education, and had a higher burden of polypharmacy, chronic disease, and physical impairment.

**Table 1:** Baseline sociodemographic, clinical and lifestyle characteristics of study sample stratified by LRTI diagnosis post propensity score matching

	No LRTI	LRTI	Total
	*N* = 1,701	*N* = 567	*N* = 2,268
Age*	78.1 (9.3)	82.6 (9.3)	79.2 (9.5)
**Sex** (female)	1,048 (61.6%)	331 (58.4%)	1,379 (60.8%)
Education*			
Elementary	272 (16.0%)	112 (19.8%)	384 (16.9%)
High school	852 (50.1%)	295 (52.0%)	1,147 (50.6%)
University	577 (33.9%)	160 (28.2%)	737 (32.5%)
**Living arrangement**			
Independent	1,624 (95.5%)	538 (94.9%)	2,162 (95.3%)
Group living	77 (4.5%)	29 (5.1%)	106 (4.7%)
**Civil status**			
Married	701 (41.2%)	210 (37.0%)	911 (40.2%)
Widowed	485 (28.5%)	191 (33.7%)	676 (29.8%)
Unmarried	278 (16.3%)	89 (15.7%)	367 (16.2%)
Divorced	237 (13.9%)	77 (13.6%)	314 (13.8%)
Alcohol intake*			
Never/occasional	621 (36.5%)	246 (43.4%)	867 (38.2%)
Light/moderate	722 (42.4%)	210 (37.0%)	932 (41.1%)
Heavy	358 (21.0%)	111 (19.6%)	469 (20.7%)
**Smoking status**			
Never	1,001 (58.8%)	351 (61.9%)	1,352 (59.6%)
Former	441 (25.9%)	131 (23.1%)	572 (25.2%)
Current	259 (15.2%)	85 (15.0%)	344 (15.2%)
**BMI** (kg/m^2^)	25.4 (4.3)	25.1 (4.2)	25.4 (4.3)
Number of drugs*	4.5 (3.4)	6.0 (4.1)	4.9 (3.7)
Asthma*	123 (7.2%)	59 (10.4%)	182 (8.0%)
Atrial fibrillation*	188 (11.1%)	129 (22.8%)	317 (14.0%)
Cerebrovascular disease*	147 (8.6%)	94 (16.6%)	241 (10.6%)
Chronic kidney disease*	617 (36.3%)	279 (49.2%)	896 (39.5%)
**Chronic liver disease**	5 (0.3%)	1 (0.2%)	6 (0.3%)
COPD*	96 (5.6%)	74 (13.1%)	170 (7.5%)
Cancer*	187 (11.0%)	114 (20.1%)	301 (13.3%)
**Diabetes**	199 (11.7%)	106 (18.7%)	305 (13.4%)
Heart failure*	183 (10.8%)	137 (24.2%)	320 (14.1%)
**Hypertension**	1,293 (76.0%)	439 (77.4%)	1,732 (76.4%)
Ischemic heart disease*	270 (15.9%)	162 (28.6%)	432 (19.0%)
Number of chronic diseases*	5.1 (2.9)	7.0 (4.0)	5.6 (3.3)
**Gait speed** (m/s)*	0.9 (0.4)	0.8 (0.4)	0.9 (0.4)
MMSE*	28.0 (2.8)	27.2 (3.5)	27.8 (3.0)
ADL*	0.7 (1.6)	1.2 (2.0)	0.8 (1.7)
IADL*	0.2 (0.6)	0.3 (0.8)	0.2 (0.7)
HAT*	7.4 (2.0)	7.0 (2.1)	7.3 (2.0)
*p-value < 0.05 Data are presented as mean (SD) for continuous measures, and n (%) for categorical measures. **Abbreviations**: ADL, activities of daily living; BMI, body mass index; COPD, chronic obstructive pulmonary disease; HAT, health assessment tool; IADL, instrumental activities of daily living; LRTI, lower respiratory tract infection; MMSE, Mini-Mental State Examination			

Due to the insufficient adjustment from the matching process (Table 1), the mixed linear regression adjusted for the confounders in the mixed-linear regression models.

### Effects of LRTI on the HAT score

**Fig. 1:**
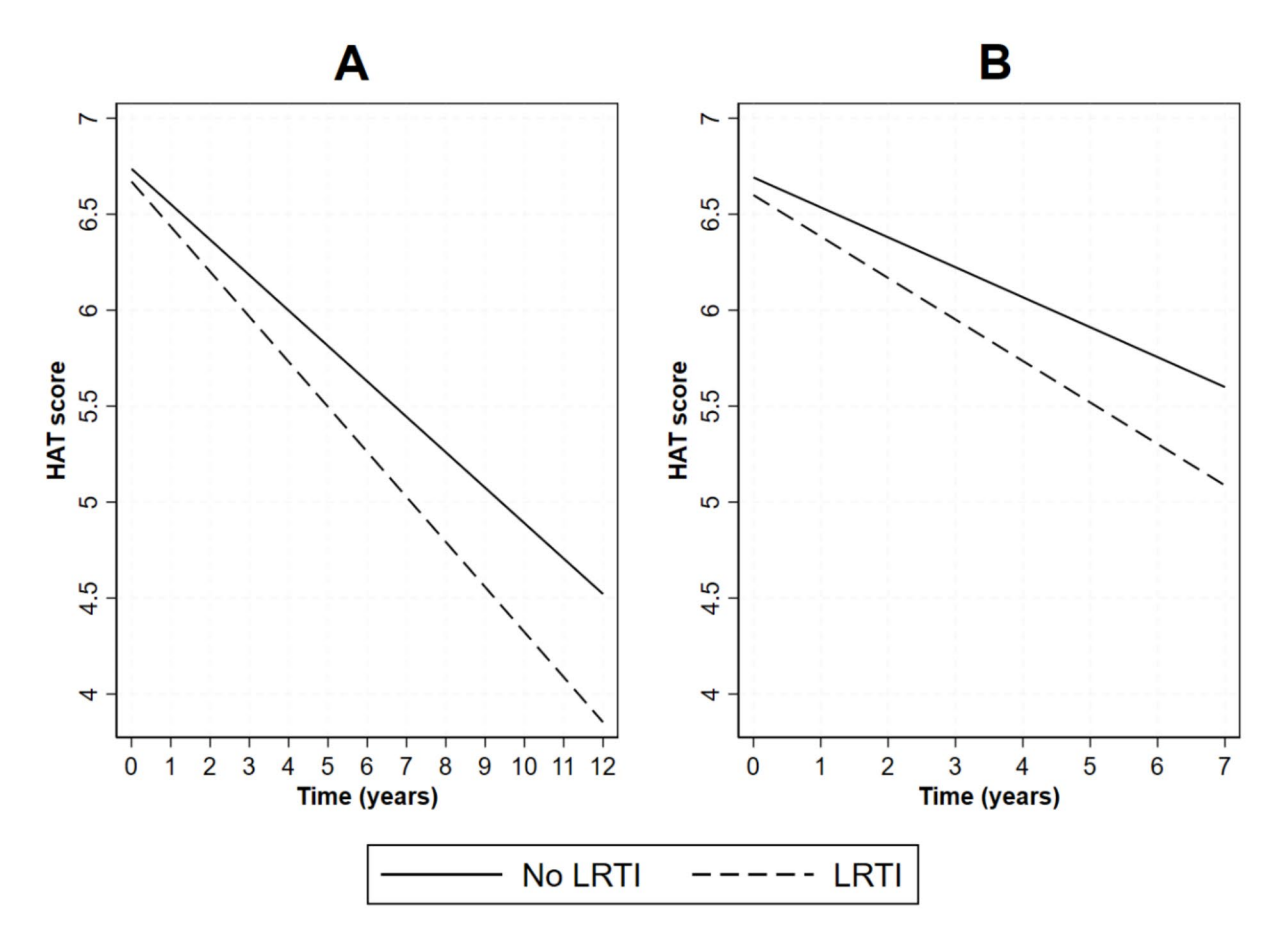
Long-term (**Panel A**) and mid-term (**Panel B**) predicted trajectories of HAT in participants with and without an LRTI diagnosis All models are adjusted for sex, age, living condition, civil status, education, smoking, alcohol consumption, body mass index, number of drugs, asthma, atrial fibrillation, cerebrovascular disease, chronic kidney disease, chronic obstructive pulmonary disease, cancer, diabetes, heart failure, hypertension, ischemic heart disease. **Abbreviations**: HAT, health assessment tool; LRTI, lower respiratory tract infection

Over the 12-year follow-up period, LRTIs were associated with an excess, though statistically non-significant, annual decline of 0.05 points in the HAT score (Table 2**)**. On the intermediate-term (i.e., up to seven years), LRTIs were associated with an excess annual decline of 0.060 (95% CI: -0.107, -0.013) points in the HAT score. Figure 1 illustrates the predicted HAT score trajectories for participants with and without an LRTI diagnosis.

**Table 2:** *β-*coefficients and 95% CIs from linear mixed effect models* exploring the association between LRTIs and annual changes in the HAT scores

	Long-term follow-up β (95% CI)		Mid-term follow-up β (95% CI)	
**Overall**				
No LRTI x time	Reference		Reference	
LRTI x time	-0.050 (-0.101 to 0.001)		-0.060 (-0.107 to -0.013)	
**Males**				
No LRTI x time	Reference		Reference	
LRTI x time	-0.097 (-0.173 to -0.021)		-0.108 (-0.177 to -0.039)	
**Females**				
No LRTI x time	Reference		Reference	
LRTI x time	-0.012 (-0.081 to 0.058)		-0.020 (-0.086 to 0.045)	

*Models are adjusted for sex, age, living arrangement, civil status, education, smoking, alcohol consumption, body mass index, number of drugs, asthma, atrial fibrillation, cerebrovascular disease, chronic kidney disease, chronic obstructive pulmonary disease, cancer, diabetes, heart failure, hypertension, ischemic heart disease

**Abbreviations**: CI, confidence interval; HAT, health assessment tool; LRTI, lower respiratory tract infections

In sex-stratified analyses, among males, LRTIs were associated with an excess annual decline in HAT scores both in the mid-term (β = -0.108 95% CI: -0.177, -0.039) and long-term (β -0.097, 95% CI: -0.173, -0.021). No statistically significant associations were observed among females.

### Sensitivity analysis

A total of 545 participants were diagnosed with pneumonia during follow-up. They were matched with 1,635 participants without a pneumonia diagnosis. Table S2 in Additional file 1 provides an overview of the baseline sociodemographic, clinical, and lifestyle characteristics of the participants stratified by pneumonia diagnosis.

Pneumonia was associated with a statistically significant excess annual decline of 0.050 (95% CI: -0.099, -0.001) points in the HAT score during the mid-term follow-up (Table 3). Similar trends were observed in the long term, although the results were not statistically significant. FigureS1 in Additional file 1 presents the predicted trajectories of HAT scores for participants with and without pneumonia exposure. Sex-stratified analyses mirrored those of LRTI, with significant associations observed only among males in both the mid-term and long-term follow-up periods.

**Table 3:** *β-*coefficients and 95% CIs from linear mixed effect models exploring the association between pneumonia and annual changes in the HAT scores

	Long-term follow-up β (95% CI)		Mid-term follow-up β (95% CI)	
**Overall**				
No pneumonia x time	Reference		Reference	
Pneumonia x time	-0.041 (-0.094 to 0.012)		-0.050 (-0.099 to -0.001)	
**Males**				
No pneumonia x time	Reference		Reference	
pneumonia x time	-0.088 (-0.167 to -0.010)		-0.095 (-0.165 to -0.025)	
**Females**				
No pneumonia x time	Reference		Reference	
pneumonia x time	-0.004 (-0.076 to 0.069)		-0.013 (-0.082 to 0.057)	

*Models are adjusted for sex, age, living arrangement, civil status, education, smoking, alcohol consumption, body mass index, number of drugs, asthma, atrial fibrillation, cerebrovascular disease, chronic kidney disease, chronic obstructive pulmonary disease, cancer, diabetes, heart failure, hypertension, ischemic heart disease

**Abbreviations**: CI, confidence interval; HAT, health assessment tool

## Discussion

This population-based cohort study aimed to investigate the mid-term and long-term effects of a single-episode of LRTIs on the objective health trajectories of older adults. Although individuals with and without an LRTI diagnosis both experienced a decline in health status with aging, those with LRTIs had a steeper annual decline. This group was estimated to have a HAT score lower by 0.42 points in 7-years, and 1.16 points in 12 years, after considering concomitant several potential confounders. Moreover, the results showed a steeper and stronger association among males, and a non-statistically significant association among females, suggesting that males are more likely to experience the longer effects of LRTI exposure compared to females.

In addition to the lingering physiological impact of infections, several other factors may contribute to an accelerated health deterioration in individuals who had LRTIs. For example, acute hospitalizations, which often accompany severe LRTI cases, have been linked to declines in both physical and mental health in older adults [36–38]. Hospitalizations for pneumonia have been specifically associated with long-term cognitive impairment [39, 40] and with onset of depressive symptoms [39]. Additionally, LRTIs are arguably an epiphenomenon or trigger of an already existing frailty substrate, which is usually accompanied by multimorbidity and escalating polypharmacy [41]. These conditions are often responsible for an increased clinical complexity and potential drug-drug and drug-disease interactions, which may also play a role in the observed accelerated health decline [42, 43].

Notably, the decline in health status following an LRTI diagnosis was statistically significant only in the mid-term follow-up, suggesting that the negative effects of LRTIs on health trajectories may be temporary, with potential partial recovery or adaptation over time. This finding aligns with a previous study conducted in the same study population as ours, which reported a temporary decline in cognitive function during the first 2.5 years after pneumonia [11]. In our study, however, we observed statistically significant differences up to seven years following LRTI diagnosis. Since HAT incorporates measures of disability, physical function, and multimorbidity in addition to cognitive function, it is possible that the non-cognitive components of HAT are the ones affected for a longer period. Although the long-term follow-up results did not reach statistical significance, we cannot rule out the presence of long-term effects given the similarity of the point estimates between mid-term and long-term follow-ups and the lower power to detect statistically significant differences arising from dropouts during the longer follow-up period.

Interestingly, the observed differential decline was only observed among males, both in shorter and longer follow-up periods. Previous studies have highlighted sex differences in LRTIs, with males being at a higher risk of contracting LRTIs and experiencing a poorer disease course [13, 15, 44]. In addition to anatomical and hormonal differences, males may also be more predisposed to respiratory infections due to their higher cardiometabolic disease burden and lifestyle (e.g., higher prevalence of smoking) [13]. Notably, several studies have reported longer LRTI-related hospital stays in males than in females, which could explain the sex differences observed in our study [15, 45]. Additionally, males have been demonstrated to have a higher risk of mortality post-LRTI [13, 15], that could last up to 16-years following a single episode of moderate-to-severe LRTI among older adults. In this regard, considering that our study design required a post-LRTI study visit to capture health status changes, it is likely that the true decline in males was even steeper than that captured in our data, due to censoring from mortality.

### Implications and future research directions

The findings of this study communicate potential implications for healthcare and public health professionals. The persistent negative effects of LRTIs beyond the acute phase highlight the importance of long-term monitoring and support for older adults, especially males, who have experienced LRTIs. Further research is needed to understand the underlying mechanisms contributing to these long-term effects, and to develop targeted interventions to optimize healthcare strategies. This study also provides indirect evidence for the importance of infection prevention measures (e.g., vaccination) as means to reduce the risk and severity of LRTIs and, consequently, minimize the long-term health decline associated with such infections. Considering that a HAT score below 5 signifies functional dependence, our results show that the prevention of an LRTI could potentially delay individuals from reaching this threshold [31]. Future studies should incorporate more recent data that contains information on COVID-19 infections as they are associated with worse outcomes among older adults, in particular those with frailty [46].

### Strengths and limitations

The strengths of the study include the use of data from a well-characterized cohort (SNAC-K) with a long follow-up period and a wealth of information about sociodemographic, lifestyle, and clinical factors. Another strength is the identification of LRTI episodes through the Swedish NPR, which possesses high completeness and captures a significant majority of cases due to the country’s universal healthcare system [26]. Additionally, the study focuses on longer-term effects rather than immediate effects during or subsequent to the infectious period which are widely studied and described.

However, several limitations of our study need to be highlighted. Firstly, the participants of SNAC-K live in an affluent urban part of Sweden, with higher-than-average attainment of university degrees compared to their age counterparts. Some potential confounders collected as part of SNAC-K study such as pack-year and forced expiratory volume at 1 s (FEV1) had many missing values in the follow-ups limiting their inclusion into the analysis. Despite the use of propensity score-based matching with an exhaustive list of covariates and further adjustment for residual potential confounding, unmeasured confounding due to factors as genetic factors and vaccination status cannot be ruled out. Nonetheless, any unmeasured confounder would need to be very strongly linked to both LRTI and health status with large effect in order to fully explain away the observed associations. Another limitation is the measurement of the HAT at fixed intervals in SNAC-K, as our assumption that the HAT remains constant between the last available measurement and the index date may not always hold true, especially for younger participants who undergo follow-up assessments less frequently. Finally, the lack of information from primary care data limits our study to moderate and severe cases of LRTIs assessed in secondary and tertiary healthcare, and excludes milder cases from the analysis. In turn, the generalizability of the results might be affected, and the outcomes might not necessarily reflect the cases of individuals who did not require medical treatment or were treated for mild infections exclusively in primary care.

## Conclusion

In summary, our study revealed the long-term effects of LRTIs on objective health trajectories of older adults. The excess health decline following LRTI diagnosis was particularly pronounced in males, among whom the negative effects may persist for up to 12 years. These findings emphasize the importance of prevention and long-term monitoring in older adults, especially males, who have experienced LRTIs.

## Electronic supplementary material

Below is the link to the electronic supplementary material.

Supplementary Material 1: Tables S1-S2, Figure S1. Table S1 – ICD-10 codes and their description, Table S2 – Baseline characteristics of the study population stratified by pneumonia status post propensity score matching, Figure S1 – health trajectories among pneumonia-exposed and -unexposed participants.

Supplementary Material 2: STROBE checklist.

## Data Availability

The datasets generated and/or analyzed during the current study are not publicly available due to ethical and data sharing restrictions/laws, including but not limited to GDPR. The data, however, can be requested formally from the SNAC-K principal investigator.

## Declarations

### Clinical trial number

Not applicable. Observational data was used.

## Abbreviations

LRTI: Lower respiratory tract infection
HAT: Health Assessment Tool
SNAC-K: Swedish National study on Aging and Care in Kungsholmen
NPR: National Patient Register
ICD-10: International Classification of Diseases, 10th revision
P-ADL: Personal activities of daily living
I-ADL: Instrumental activities of daily living
MMSE: Mini-mental state examination
BMI: Body mass index
COPD: Chronic obstructive pulmonary disease
CI: Confidence interval
STROBE: Strengthening the reporting of observational studies in epidemiology
