# Supplementary material for: Sex differences in the impact of lower respiratory tract infections on older adults’ health trajectories: a population-based cohort study
Source: BMC Infect Dis. 2024 Nov 1;24:1227. doi: 10.1186/s12879-024-10131-7 (PMC11529179; doi:10.1186/s12879-024-10131-7)
Supplement: Supplementary file 2 — Supplementary Material 2: STROBE checklist. [file 12879_2024_10131_MOESM2_ESM.docx]

**Sex differences in the impact of lower respiratory tract infections on older adults’ health trajectories: a Swedish population-based cohort study**

**Additional File 1**

**Journal: BMC Infectious Diseases**

**Authors**

Ahmad Abbadi*^1,2^, Giorgi Beridze*^1^, Eleana Tsoumani^3^, Agnes Brandtmüller^4^, Merle K Hendel^1^, Stina Salomonsson^5^, Amaia Calderón-Larrañaga^1,6^, Davide L. Vetrano^1,6^

* Abbadi and Beridze contributed equally to the manuscript, and are co-first authors.

**Affiliations**

1. Aging Research Center, Department of Neurobiology, Care Sciences and Society, Karolinska Institutet and Stockholm University, Stockholm, Sweden
2. Department of Medical Epidemiology and Biostatistics, Karolinska Institutet, Solna, Sweden
3. Center for Observational and Real-World Evidence, MSD, Athens, Greece
4. Center for Observational and Real-World Evidence, MSD, Budapest, Hungary
5. Center for Observational and Real-World Evidence, MSD, Stockholm, Sweden
6. Stockholm Gerontology Research Center, Stockholm, Sweden

**Corresponding author**

Ahmad Abbadi, MD MMedSc

Department of Medical Epidemiology and Biostatistics, Karolinska Institutet, Solna, Sweden

Nobels väg 12A, 171 65 Solna, Sweden

[ahmad.abbadi@ki.se](mailto:ahmad.abbadi@ki.se)

**Table S1**. ICD-10 codes used in identification of LRTI and their description

| ICD-10 Code | Description |
| --- | --- |
| J09 | Influenza due to certain identified influenza viruses |
| J10 | Influenza due to other identified influenza virus |
| J11 | Influenza due to unidentified influenza virus |
| J12 | Viral pneumonia, not elsewhere classified |
| J13 | Pneumonia due to Streptococcus pneumoniae |
| J14 | Pneumonia due to Hemophilus influenzae |
| J15 | Bacterial pneumonia, not elsewhere classified |
| J16 | Pneumonia due to other infectious organisms, not elsewhere classified |
| J17 | Pneumonia in diseases classified elsewhere |
| J18 | Pneumonia, unspecified organism |
| J20 | Acute bronchitis |
| J21 | Acute bronchiolitis |
| J22 | Unspecified acute lower respiratory infection |

**Table S2.** Baseline sociodemographic, clinical and lifestyle characteristics of study sample stratified by pneumonia diagnosis post propensity score matching

|  | **No pneumonia** | **Pneumonia** | **Total** |
| --- | --- | --- | --- |
|  | **N=1,635** | **N=545** | **N=2,180** |
| **Age*** | 78.6 (9.2) | 82.7 (9.2) | 79.6 (9.4) |
| **Sex** (female) | 1,005 (61.5%) | 315 (57.8%) | 1,320 (60.6%) |
| **Education*** |  |  |  |
| Elementary | 272 (16.6%) | 105 (19.3%) | 377 (17.3%) |
| High school | 819 (50.1%) | 284 (52.1%) | 1,103 (50.6%) |
| University | 544 (33.3%) | 156 (28.6%) | 700 (32.1%) |
| **Living arrangement** |  |  |  |
| Independent | 1,559 (95.4%) | 518 (95.0%) | 2,077 (95.3%) |
| Group living | 75 (4.6%) | 26 (4.8%) | 101 (4.6%) |
| **Civil status** |  |  |  |
| Married | 670 (41.0%) | 203 (37.2%) | 873 (40.0%) |
| Widow/er | 483 (29.5%) | 183 (33.6%) | 666 (30.6%) |
| Unmarried | 260 (15.9%) | 85 (15.6%) | 345 (15.8%) |
| Divorced | 222 (13.6%) | 74 (13.6%) | 296 (13.6%) |
| **Alcohol intake*** |  |  |  |
| Never/occasional | 617 (37.7%) | 235 (43.1%) | 852 (39.1%) |
| Light/moderate | 682 (41.7%) | 200 (36.7%) | 882 (40.5%) |
| Heavy | 336 (20.6%) | 110 (20.2%) | 446 (20.5%) |
| **Smoking status** |  |  |  |
| Never | 971 (59.4%) | 338 (62.0%) | 1,309 (60.0%) |
| Former | 416 (25.4%) | 125 (22.9%) | 541 (24.8%) |
| Current | 248 (15.2%) | 82 (15.0%) | 330 (15.1%) |
| **BMI** (kg/m^2^) | 25.4 (4.3) | 25.0 (4.2) | 25.3 (4.3) |
| **Number of drugs*** | 4.6 (3.5) | 6.0 (4.2) | 5.0 (3.7) |
| **Asthma*** | 121 (7.4%) | 54 (9.9%) | 175 (8.0%) |
| **Atrial fibrillation*** | 190 (11.6%) | 125 (22.9%) | 315 (14.4%) |
| **Cerebrovascular disease*** | 148 (9.1%) | 92 (16.9%) | 240 (11.0%) |
| **Chronic kidney disease*** | 615 (37.6%) | 271 (49.7%) | 886 (40.6%) |
| **Chronic liver disease** | 6 (0.4%) | 2 (0.4%) | 8 0.4%) |
| **COPD*** | 97 (5.9%) | 74 (13.6%) | 171 (7.8%) |
| **Cancer*** | 187 (11.4%) | 110 (20.2%) | 297 (13.6%) |
| **Diabetes** | 196 (12.0%) | 104 (19.1%) | 300 (13.8%) |
| **Heart failure*** | 186 (11.4%) | 135 (24.8%) | 321 (14.7%) |
| **Hypertension** | 1,247 (76.3%) | 424 (77.8%) | 1,671 (76.7%) |
| **Ischemic heart disease*** | 271 (16.6%) | 158 (29.0%) | 429 (19.7%) |
| **Number of chronic diseases*** | 5.2 (3.0) | 7.1 (4.1) | 5.7 (3.4) |
| **Gait speed** (m/s)* | 0.9 (0.4) | 0.8 (0.4) | 0.9 (0.4) |
| **MMSE*** | 27.9 (2.9) | 27.2 (3.5) | 27.7 (3.1) |
| **ADL*** | 0.7 (1.6) | 1.2 (2.1) | 0.8 (1.8) |
| **IADL*** | 0.2 (0.6) | 0.3 (0.8) | 0.2 (0.7) |
| **HAT*** | 7.3 (2.0) | 7.0 (2.1) | 7.2 (2.1) |
| *p-value < 0.05  Data are presented as mean (SD) for continuous measures, and n (%) for categorical measures.  **Abbreviations**: ADL, activities of daily living; BMI, body mass index; COPD, chronic obstructive pulmonary disease; HAT, health assessment tool; IADL, instrumental activities of daily living; MMSE, Mini-Mental State Examination | | | |

**Figure S1.** Long-term (**Panel A**) and mid-term (**Panel B**) predicted trajectories of the HAT in participants with and without a pneumonia diagnosis

*
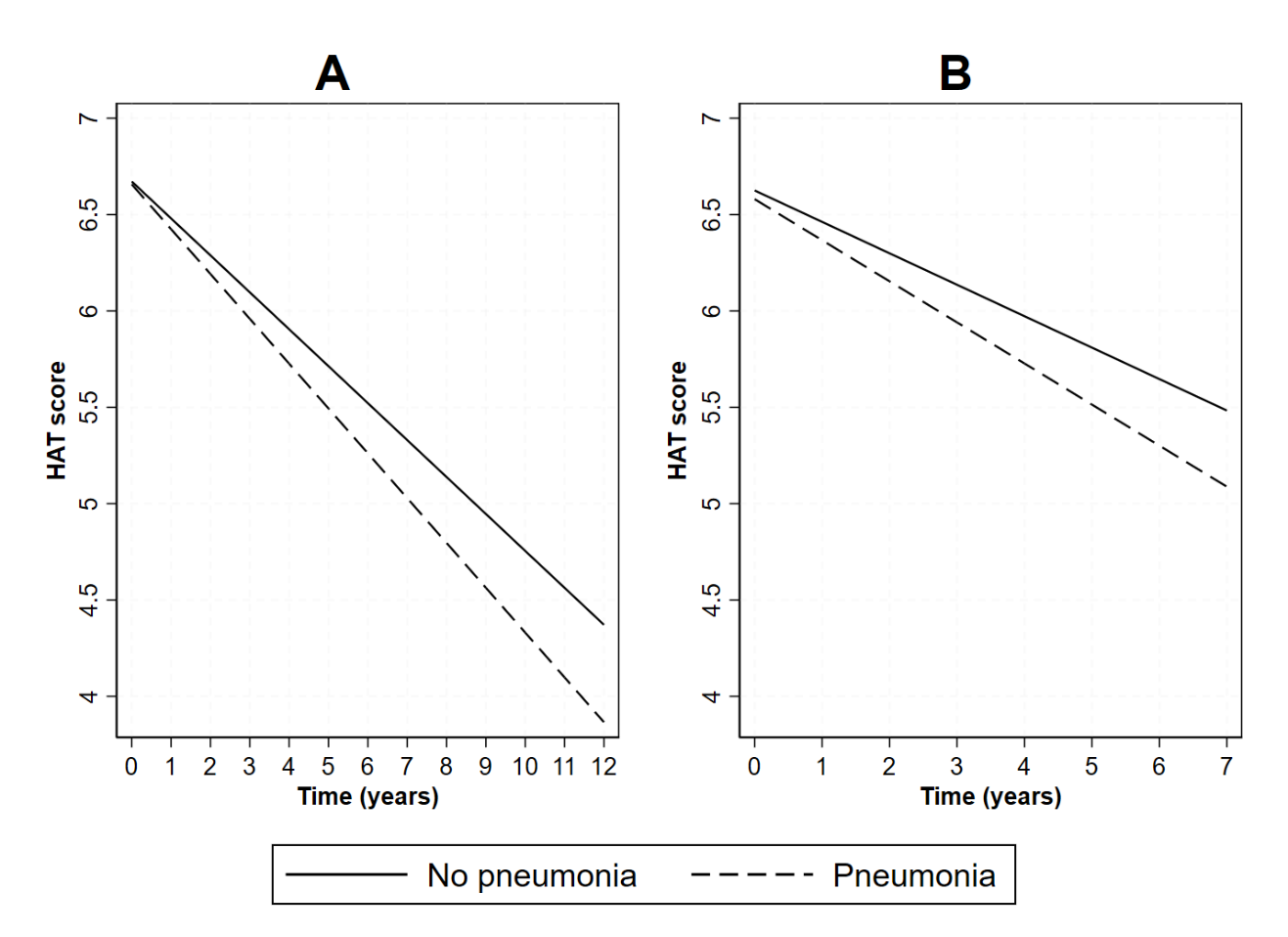
*

All models are adjusted for sex, age, living arrangement, civil status, education, smoking, alcohol consumption, body mass index, number of drugs, asthma, atrial fibrillation, cerebrovascular disease, chronic kidney disease, chronic obstructive pulmonary disease, cancer, diabetes, heart failure, hypertension, ischemic heart disease.

**Abbreviations**: HAT, health assessment tool;
